# Chloroform

**Published:** 1896-03

**Authors:** Thos. R. Evans

**Affiliations:** Mt. Carbon, W. Va.


### CHLOROFORM.

BY THOS. R. EVANS, M. D., MT. CARBON, W. VA.

Chloroform will be used less often in the future. The hypodermic anæsthesia and the cumbrous apparatus for giving oxygen with chloroform tend to lessen its usefulness and popularity.

These oxygen apparatus are as superfluous as Sir Isaac Newton's method was for the ingress and egress of his cat and kittens—a big hole for the cat and a little hole for the kittens. It is well known that it is safer to administer chloroform out of doors, but this does not necessitate the manufacture of oxygen in the house. The atmosphere indoors and out of doors contains all the oxygen necessary for the proper giving of chloroform. A man who is accustomed to improperly give ether is a most unsafe man to administer chloroform. Without knowing it, he gives ether on the principle that a man can live under water. A patient, said to be etherized by him, has his animation suspended as if by drowning, ether containing the same amount of oxygen as is contained in water. Only the mechanical effects of water are absent. The struggling under ether has been too much attributed to its stimulative effects.

I am more and more impressed with the fact that a cone with a large opening in its apex is the safest mode of administering chloroform anæsthesia. A few days ago a man lost his life from chloroform in this state. He had twice been under chloroform, which would seem to exclude the overrated idiosyncrasy excuse, etc. It is not known what form of apparatus were previously used, but he died under a copper frame with supposable porous material tightly stretched over it, upon which the chloroform was dropped. The old verdict might have been pronounced in this case: Died from want of breath. Doubtless, the cold and heavy anæsthetic had closed the pores in the gauze. The doctors were as careful as the average, and the operation, straightening of a flexed finger, had not been begun.

There should be a chair of physics as well as of physic, in the medical colleges. Atmospheric air is a peculiar mixture. It was thought that Priestley had completed its analysis over a hundred years ago. Now two men (they are not M. D.'s) join hands over argon.

It is not altogether imagination that during a paroxysm, the asthmatic cannot breathe so well under mosquito netting. Notwithstanding quite thorough ventilation, the same air will stay in the corner of a room for days and days.

I would recommend a method, with hope, for restoring from too profound anæsthesia. It is recommended to take the place of stretching the anus. The introduction of a catheter into the bladder. It is well known that there is a reflex center of respiration in the genitalia. This is shown in certain stages of coition, and is probably independent of the sphincteric reflex. The genitals are the last portions to lose sensibility, and a catheter in the bladder is the nearest possible approach to the seat or basin of the great sympathetic. No harm can be done with a clean catheter, while much harm may be done to the anus under excitement.

Since writing the above I have learned two things: first, that a cone made in the usual way was used in the above case, but that the middle of its apex was pinned, leaving an opening on each side of the pin; second, that either the Esmarch inhaler described above, or German chloroform is a very dangerous thing. For, in Germany, in 1894, according to collective investigation, there was the most appalling number of twenty-three (23) deaths from chloroform narcosis out of 31,843 administrations. Considering the fertility of the Germans in chemical technology, the fault is either in the mode of administration, or possibly in the climate.

Such numerous fatalities would banish its use from southern climates. I think there is no doubt that chloroform is most safely given in the warm months, when the air is most highly oxygenated. The natural pallidness of the German race and its tuberculous diathesis show a lack of oxygen.

